# Eukaryotic elongation factor 2 is a prognostic marker and its kinase a potential therapeutic target in HCC

**DOI:** 10.18632/oncotarget.14447

**Published:** 2017-01-02

**Authors:** Leona L Pott, Sascha Hagemann, Henning Reis, Kristina Lorenz, Thilo Bracht, Thomas Herold, Boris V Skryabin, Dominik A Megger, Julia Kälsch, Frank Weber, Barbara Sitek, Hideo A Baba

**Affiliations:** ^1^ Institute of Pathology, University of Duisburg-Essen, Essen, Germany; ^2^ Medizinisches Proteom-Center, Ruhr-University Bochum, Bochum, Germany; ^3^ Institute of Pharmacology, University of Wuerzburg, Wuerzburg, Germany; ^4^ Leibniz-Institut für Analytische Wissenschaften –ISAS-e.V., Dortmund, Germany; ^5^ West German Heart and Vascular Center, University of Duisburg-Essen, Essen, Germany; ^6^ Transgenic Animal and Genetic Engineering Models (TRAM), Westphalian Wilhelms University, Muenster, Germany; ^7^ Department of Gastroenterology and Hepatology, University of Duisburg-Essen, Essen, Germany; ^8^ Department of General, Visceral and Transplantation Surgery, University of Duisburg-Essen, Essen, Germany

**Keywords:** eEF2, eEF2 kinase, prognosis, hepatocellular carcinoma

## Abstract

Hepatocellular carcinoma is a cancer with increasing incidence and largely refractory to current anticancer drugs. Since Sorafenib, a multikinase inhibitor has shown modest efficacy in advanced hepatocellular carcinoma additional treatments are highly needed. Protein phosphorylation via kinases is an important post-translational modification to regulate cell homeostasis including proliferation and apoptosis. Therefore kinases are valuable targets in cancer therapy. To this end we performed 2D differential gel electrophoresis and mass spectrometry analysis of phosphoprotein-enriched lysates of tumor and corresponding non-tumorous liver samples to detect differentially abundant phosphoproteins to screen for novel kinases as potential drug targets. We identified 34 differentially abundant proteins in phosphoprotein enriched lysates. Expression and distribution of the candidate protein eEF2 and its phosphorylated isoform was validated immunohistochemically on 78 hepatocellular carcinoma and non-tumorous tissue samples. Validation showed that total eEF2 and phosphorylated eEF2 at threonine 56 are prognostic markers for overall survival of HCC-patients. The activity of the regulating eEF2 kinase, compared between tumor and non-tumorous tissue lysates by *in vitro* kinase assays, is more than four times higher in tumor tissues. Functional analyzes regarding eEF2 kinase were performed in JHH5 cells with CRISPR/Cas9 mediated eEF2 kinase knock out. Proliferation and growth is decreased in eEF2 kinase knock out cells.

**Conclusion:**

eEF2 and phosphorylated eEF2 are prognostic markers for survival of hepatocellular carcinoma patients and the regulating eEF2 kinase is a potential drug target for tumor therapy.

## INTRODUCTION

Hepatocellular carcinoma (HCC) is the most frequent malignant liver cell tumor and based on its high lethality it represents the third to fourth numerous cause of death concerning tumor diseases worldwide. It is the sixth most common neoplasm with increasing incidence [1, 2]. Most cases of HCC are based on chronic liver diseases like viral hepatitis or alcohol abuse [3]. As symptoms occur in advanced phases of the disease, HCC commonly is detected at late stages and most of the patients die within one year after diagnosis. One main reason for poor prognosis is the lack of curative treatment. One admitted therapy option is the multikinase inhibitor Sorafenib, showing modest efficacy in advanced HCC with frequently occurring side effects [4]. Thus, additional treatments are highly needed and protein kinases came to the fore because phosphorylation and dephosphorylation of proteins are involved in many cellular processes, e.g. metabolism, cellular signaling, proliferation, and cell survival and control several biological responses such as differentiation, invasion, metastasis and apoptosis [5, 6]. Changes in phosphorylation patterns can be either the consequence or the cause of many diseases, e.g. cancer [7, 8]. As a consequence regulation of signaling pathways by the responsible kinases is of particular interest to find new therapeutically relevant drug targets [9–11]. Therefore, in this study we initially compared phosphoprotein-enriched lysates of HCC tissue and corresponding non-tumorous liver samples of 7 HCC-patients in a classical phosphoproteomic approach using 2D DIGE. Three isoforms of eEF2 were identified to be differentially abundant in HCC. In further studies, we could show that expression of total eEF2 and eEF2 phosphorylated at threonine 56 has a prognostic value for patients’ overall survival and that the activity of eEF2 kinase, responsible for the phosphorylation of eEF2, is significantly increased in HCC-tissues. To investigate the influence of the eEF2 kinase on tumor cell growth *in vitro* we performed a CRISPR/Cas9 mediated knock out in the HCC cell line JHH5 and studied the effects on growth and proliferation.

## RESULTS

### 34 identified proteins showed differential abundance in phosphoprotein-enriched lysates of non-tumorous and HCC-liver tissues

**Figure 1:**
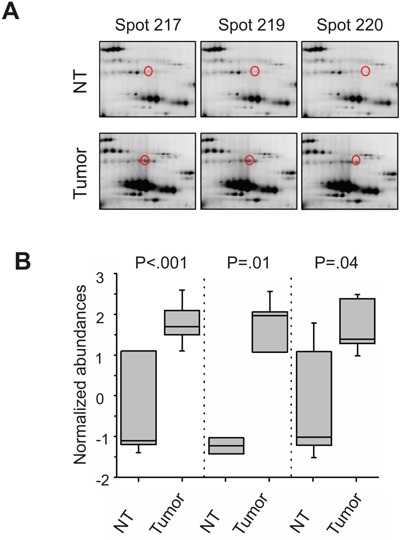
2D DIGE analysis with enriched phosphoproteins of HCC tissue lysates Representative spot patterns from 2D DIGE analysis from one HCC-patient. eEF2(marked in red) in non-tumorous (NT) and corresponding HCC (tumor) liver tissue lysate, enriched for phosphoproteins of one HCC-patient **A, B**. Boxplots with the normalized abundances of the three eEF2 -isoforms of all 7 HCC-patients, which were analyzed in this study. Boxes represent 25th and 75th percentiles; whiskers indicate highest and lowest value. The median is shown as a horizontal line, and the mean value as a square within the box.

Within the 2D DIGE study of phosphoprotein-enriched lysates from seven HCC-patients we identified 57 protein spots that were differentially abundant in non-tumorous and tumor tissue of the patients. Criteria for a differential abundance were a 1.5-fold over- or underrepresentation and a statistical p-value of less than .05. After mass spectrometric protein identification via MALDI-TOF-MS peptide mass fingerprinting these 57 protein spots could be addressed to 34 different proteins (Supplementary Table 1). In tumor tissue 12 proteins were over- und 22 underrepresented. According to fold change and p-value cut off criteria, the appearance in more than one spot on the gel and being a known phosphoprotein, we chose three of these proteins for initial further validation (Figure 1), namely: heat shock cognate protein 71 (HSC70), Moesin, and eukaryotic elongation factor 2 (eEF2). For gel images and normalized expression of HSC70 and Moesin please see supplementary figure 1. “In dephosphorylation and off gel fractionation experiments eEF2 appeared to be generally phosphorylated but showed different phosphorylation patterns in HCC tissue compared to non-tumorous tissue (Supplementary Figure 2).

### Expression of total eEF2 and peEF2(T56) is a prognostic marker for overall survival of HCC-patients

**Figure 2:**
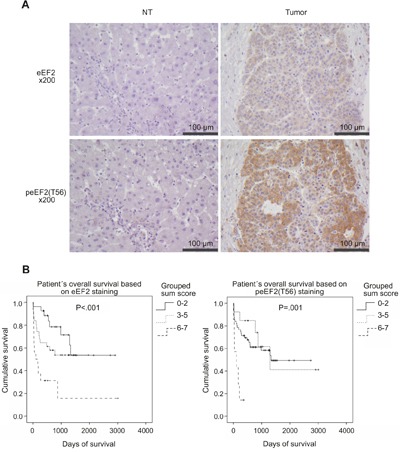
Immunohistochemical evaluation of eEF2 and peEF2(T56) on TMAs of HCC-patients TMAs of 78 HCC patients were stained against eEF2 and peEF2(T56) Representative staining in non-tumorous and tumor tissues is shown in **A**. Figure 2 **B**. shows the overall survival of HCC-patients based on a sum score of number of eEF2 and peEF2(T56) positive cells and staining intensity in a tumor section. Sum scores were grouped to 0-2, 3-5 and 6-7. Patients harboring HCC with a high number of positive cells combined with high intensity have a significant shorter survival time.

To validate the overexpression tissue micro arrays (TMAs) of an initial training set of 16 HCC-patients were immunohistochemically stained against eEF2, HSC70 and Moesin. After staining evaluation HSC70, Moesin and pMoesin (phospho-Ezrin-Radixin-Moesin) did not show differences between HCC and non-HCC-tissues (data not shown). Therefore we decided to further validate eEF2 only. In the validation set, TMAs of further 78 HCC-patients (Table 1) were immunohistochemically stained against eEF2. A representative comparison of the eEF2 staining of non-tumorous and HCC tissue is shown in Figure 2A. Evaluation of eEF2 expression alone and combined with intensity detected a significant increase in tumor tissue (*P*<.001). Patients with a high combined score of eEF2 positive cells and staining intensity had significantly shorter overall survival rate (*P*<.001; Figure 2B). We further stained the TMAs of the HCC-patients against peEF2(T56) (example is shown in Figure 2A). The evaluation (Figure 2B, right graph) showed a prognostic value in R0-resected HCC-patients (*P*=.001). TMAs for immunohistochemical evaluation did include patients in which HCC was based on HBV/HCV-infection (Table 1). There was no correlation of HBV/HCV- infection with number and staining intensity of eEF2 or peEF2(T56).

**Table 1:** General clinico-pathological data of the HCC-cohort used in this study (* One patient tissue sample without information in training set, ^#^ patients are included in IHC sets)

		Fresh frozen tissue for proteome-based studies^#^	Training Set	Validation Set
Sex	Male	5	13	57
	Female	2	3	21
	Total	7	16	78
Age mean in years (SD)		62(10)	57(17)	62(16)
HBV/HCV		1	N.N.	12
Stage*	pT1	3	11	32
	pT2	3	4	28
	pT3	1	-	15
	pT4	-	-	3
Lymphnode status*	pN0	7	14	74
	pN1	-	1	4
Grading*	G1	1	4	10
	G2	4	7	42
	G3/G4	2	4	26
Lymphvessel status*	L0	7	14	78
	L1	-	1	0
Bloodvessel status*	V0	6	12	44
	V1	1	3	34
Margins*	R0	7	15	64
	R1/R2	-	-	14

### peEF2(T56) is significantly increased in HCC-patients

**Figure 3:**
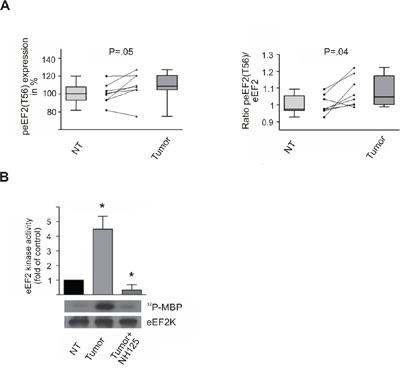
eEF2 kinase activity is increased in HCC tissue samples peEF2(T56) expression and eEF2 kinase activity in non-tumorous (NT) and corresponding HCC tissue (Tumor) assayed by immunoblotting **A**. Boxplots show the alteration of expression of peEF2(T56) in HCC tissue compared to non-tumorous tissue based on densiometric analyses of immunoblots (left panel). The ratio of peEF2(T56) to total eEF2 level shows a statistically significant increase 108% (*P*=.036) in tumor in comparison to non-tumorous cells. Boxes represent 25th and 75th percentiles; whiskers indicate the standard deviation. The median is shown as a horizontal line, and the mean value as a square within the box. The lines interconnect the corresponding HCC and NT tissue of single patients. **B**. EEF2 kinase activity was determined by *in vitro* kinase assay of non-tumorous and HCC-liver lysates. In tumor lysates the eEF2 kinase activity was significantly increased compared to non-tumorous lysates. As a control tumor lysates were treated with the eEF2 kinase inhibitor NH125 (3μM), which abolished kinase activity nearly completely. Activity of non-tumorous lysates was set to 100%. Immunoblots against immunoprecipitated eEF2 kinase confirmed that equal amounts of the kinase were put into the assay. * *P*<.01, all data are mean ± SE.

In order to confirm the immunohistochemical data and see whether peEF2(T56) alone is overrepresented in HCC we investigated total protein lysates of non-tumorous and HCC liver tissues for total eEF2 and peEF2(T56) level by immunoblotting. Compared to the non-tumorous situation in HCC-lysates we could observe an increase of peEF2 to 109% as compared to non-tumorous level (*P*=.05; Figure 3A). Comparing the ratio of peEF2 to total eEF2 in HCC-lysates revealed a statistically significant increase to 108% (*P*=.036; Figure 3A).

### HCC tumor tissues show significantly higher eEF2 kinase activity

Phosphorylation of threonine 56 residue by eEF2 kinase results in an inhibition of eEF2. Based on the immunohistochemical and western blot data we decided to investigate whether there are differences in eEF2 kinase (eEF2K) activity. *In vitro* kinase-assays revealed a 4-5 times higher eEF2K activity in tumor tissue lysates as compared to non-tumorous liver lysates (Figure 3B). Non-specific phosphorylation was determined by incubation with the eEF2K-specific inhibitor NH125 (3μM).

To rule out that these activity alteration is of genetical origin, NGS was performed with 24 tumor tissue samples and corresponding non-tumorous tissue. Single nucleotide polymorphisms (SNPs) found in the untranslated region were not tumor specific. Furthermore no SNPs could be detected in the kinase domain. The data is available at European Nucleotide Archive with the accession number PRJEB14915 (http://www.ebi.ac.uk/ena/data/view/PRJEB14915).

### EEF2 kinase knock out leads to a decreased growth rate and changes in cell morphology

**Figure 4:**
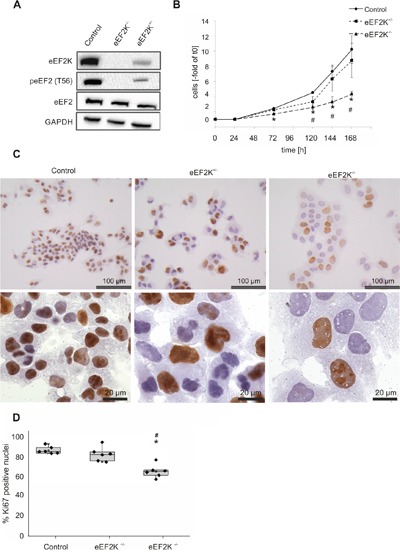
eEF2 kinase knock out in HCC cell lines leads to changes in proliferation **A**. Immunoblotting against eEF2K, peEF2 and eEF2 (GAPDH as loading control). In eEF2K^-/−^ cells no phosphorylated eEF2 is detectable. eEF2K^+/−^ cells show less kinase expression and therefore less eEF2 phosphorylation. **B**. Growth curves expressed as fold change of initially seeded cells for the three cell lines. Three independent experiments were carried out. * *P*<.01 eEF2K^-/−^ compared to control, ^#^*P*<.01 eEF2K^+/−^ compared to control, the error bars indicate ±SD. **C**. Representative immunohistochemical Ki67 staining is shown. Of note the increased size of nuclei in eEF2K^-/−^ and eEF2K^+/−^ cells compared to controls. **D**. Boxplots with single data points illustrate the percentage of Ki67 positive nuclei. 120 nuclei were evaluated in six independent experiments respectively. In eEF2K^-/−^ cells significantly less nuclei are stained compared to control cells (* *P*<.01) and eEF2K^+/−^ (^#^ *P*<.01) (Control: 84%, eEF2K^-/−^ 68%, eEF2K^+/−^: 81%).

To identify potential changes in HCC cell line proliferation and growth, we knocked out the eEF2 kinase via the CRISPR/Cas9 system in JHH5 cells. The absence of eEF2K was verified via immunoblotting for eEF2 kinase and its phosphorylation product peEF2(T56). In one single cell clone expanded to populations no eEF2 kinase and no phosphorylated eEF2 was detectable (Figure 4A). One clone showed reduced expression and decreased activity. TOPO cloning and subsequent sequencing of the single PCR fragments revealed in case of eEF2K^+/−^ one allele has a 16 bp deletion leading to a frameshift and premature stop codon and the second allele has a deletion of 3 bp, leading to deleted residue asp177, and hence to a functional protein.

Growth curve experiments were normalized to confluence and exhibited an increased doubling time for eEF2K^-/−^ cells (Control cells: 46 h ± 6h; eEF2K^-/−^ 62 h ± 6h; *P*=.02). The calculated doubling time of eEF2K^+/−^ cells is merely slightly increased (51h ± 8h). Increment of cells related to initial cell number is shown in Figure 4B. Immunohistochemical staining of the proliferation marker Ki67 showed significantly less proliferative cells (84 % positively stained nuclei in control cells, 68 % positive nuclei in eEF2K^-/−^; *P*=.002), while eEF2K^+/−^ cells have a more similar proliferation rate compared to control cells (81 %, *P*=.1). (Figure 4D).

**Figure 5:**
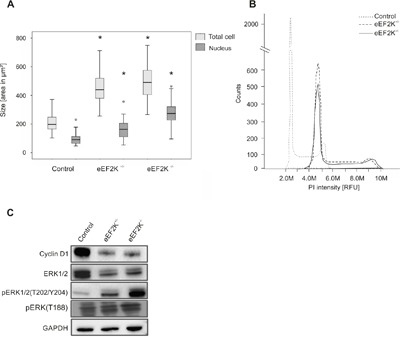
EEF2K^-/−^ cells show alterations in morphology, DNA content and protein expression **A**. Boxes represent cell size and nucleus size of eEF2K^-/−^ and eEF2K^+/−^ cells are significantly increased compared to controls, *P<.01. 100 cells and nuclei were analyzed and boxes represent 25th and 75th percentiles; whiskers indicate the standard deviation. The median is shown as a horizontal line, and the mean value as a square within the box. **B**. Flow cytometric analysis of PI stained nuclei is shown. The histogram shows a peak shift to higher propidiumiodid fluorescence intensities occurring in eEF2K knock out cells indication increased DNA content. **C**. Representative immunoblots of control cells, eEF2K^+/−^ and eEF2K^-/−^ cells stained with antibodies against Cyclin D1, ERK1/2 and pERK1/2(T202/Y204) and GAPDH as loading control. The antibody against pERK(T188) detects ERK1/2 phosphorylation at T188 and ERK2 phosphorylation at T208. eEF2K-/− cells express less Cyclin D1 and show increased phosphorylation of three ERK phosphorylation sites while total ERK1/2 is less expressed.

For morphologic studies and further comparison of proliferation the cells were grown on chamber slides for 24 hours and directly formalin fixed on the slides. Fixation and staining revealed a significant 2,3-fold (*P*<.001) enlargement of eEF2K^-/−^ cells (500 μm^2^ ± 129 μm^2^ compared to 217 μm^2^ ± 68 μm) while the mean area of eEF2K^+/−^ cells was two times higher compared to control cells (425 μm^2^ ± 104μm^2^) (Figure 5A). This was paralleled by an increase of nuclei size. While control nuclei have a mean area of 99 μm^2^ ±36 μm^2^, eEF2K^-/−^ cells show an increased area of 267 μm^2^ ± 78 μm^2^. Nuclear size of eEF2K^+/−^ is increased 1.6 times (166 μm^2^ ± 67 μm^2^) (Figure 5A). Since the main reason for enlarged nuclei is an increase in DNA, as a rule caused by polyploidization we determined the DNA content and analyzed the cell cycle distribution using flow cytometry of propidium iodide (PI) stained cells. The mean PI fluorescence intensity is 2,4x 10^6^ RFU in stained control nuclei while it is 4,4x 10^6^ RFU and 4×10^6^ RFU for eEF2K^-/−^ and eEF2K^+/−^ nuclei, respectively (Figure 5B). Distribution of cell cycle phases revealed a marginal cell cycle delay at G_1_ (59 % of cells) as shown in Figure 5B. Western blot analysis exhibits a decrease of Cyclin D1 expression in eEF2K^-/−^ and eEF2K^+/−^ cells at the same level as well as an increase in phosphorylation of ERK1/2 at residues threonine 202, tyrosine 204 and threonine 188 as shown in Figure 5C.

## DISCUSSION

The average number of spots we detected comparing the enriched phosphoproteins of 7 HCC-patients was in a similar range of resolution compared to other gel-based phosphoproteomic studies [12–15]. Comparison of untreated and dephosphorylated protein lysates after off-gel fractionation could point to different phosphorylation states as eEF2 is not only phosphorylated at regulating threonine 56 by the eEF2 kinase but also at serine 595 by Cyclin A-Cyclin-Dependent Kinase 2 serving as a “docking” phosphorylation for eEF2 kinase [16, 17]. Evaluating immunohistochemical staining of eEF2 and peEF2(T56) showed that this protein and its phosphorylation are highly significant prognostic markers for overall survival of HCC-patients. High eEF2 levels have been suggested to be associated with poor prognosis in hormone receptor-positive breast cancer [18]. Additionally, eEF2 was found to be overexpressed in gastrointestinal cancers promoting G2/M progression and enhancing cell growth *in vitro* and *in vivo* [19]. An overexpression of eEF2 is already known from several transformed cell lines and also in HCC [19–22], but to our knowledge this study for the first time describes a prognostic value of eEF2 and peEF2(T56) expression for HCC.

In the process of protein translation, eEF2 plays an important regulatory role. Being phosphorylated by eEF2 kinase at threonine 56 eEF2 loses its activity resulting in a decreased or even completely suppressed protein biosynthesis [23]. This regulation is essential for survival once the cell has entered a state of lacking energy. The microenvironment of solid tumors is characterized by hypoxia and one important step in progression to malignant disease seems to be the successful overcoming of translational regulation during hypoxia [24–27]. As high activity and levels of eEF2 kinase are thought to be the basis for tumors to become resistant to nutrient deprivation [29] we checked whether we could find increased eEF2 kinase activity in HCC-lysates. *In vitro* kinase assays of immunoprecipitated eEF2 kinase indeed showed a 4-5 times higher activity in tumor lysates compared to the corresponding non-tumorous liver lysates. Several transformed cell lines overexpress eEF2 and eEF2 kinase, which in turn leads to elevated levels of phosphorylated eEF2 [19–21]. Thus, elevated activity of eEF2 kinase in HCC-tissues might be a rational explanation for the observed increased levels of phosphorylated eEF2 in these tissues. Little is known about the regulation of expression and activity of eEF2 kinase. eEF2 kinase knockout mice are vital and fertile showing no obvious differences to wildtype littermates [30]. Regulation of activity seems to be a complicated mix of autophosphorylation in combination with additional activating or inhibiting phosphorylations [23]. Studies correlating downregulation of the eEF2 kinase with decreased levels of, e.g. Bcl-2 and Cyclin D1, suggest that eEF2 kinase directly or indirectly inhibits signal pathways that are associated with transformation [31]. Besides eEF2 and eEF2 kinase were found to be possible drug targets in gastrointestinal and breast cancer [19, 31]. Wang et al. reported a tumor suppressing activity of the herbal formulation *Huanglian Jiedu Decoction* by activating the eEF2 kinase and thus inhibiting eEF2 in HCC [32]. Contrary, Tekedereli and colleagues reported that a siRNA-silencing of eEF2 kinase resulted in growth inhibition of tumor cells [31]. Since siRNA knockdown usually does not lead to the total absence of a protein we used the CRISPR/Cas9 technique to knock out the kinase. Interestingly, a biallelic knock out with only less expression and activity of the kinase shows the same morphological changes but does not decrease the proliferation rate of HCC cells, while a total absence of the kinase results in a significant decrease of doubling time and proliferation discovered by Ki67, even without lacking energy. These data indicate that the eEF2 kinase itself or through negative feedback mechanisms could be involved in broader cellular pathways than described until now.

Confusing is the fact that the absence of this kinase which is only reported as the inhibitor of eEF2 leads to a vast increase of the size. However, a knock out of the eEF2 kinase leads to an increased translation rate by eEF2 activation. The interaction between mass accumulation and cell division is not fully understood but general opinion is that they are completely separate processes [33]. Potentially, a higher translation rate accompanied by less proliferation leads to a size increment. Recently, a newly identified autophosphorylation site of ERK2 was reported to be involved in cardiac hypertrophy [34]. In eEF2K^-/−^ cells phosphorylation of this residue is highly increased. Brietz et al. hypothesized pERK2(T188) phosphorylates additional targets located at the nucleus and promote the hypertrophic response [35]. The along going increase of nuclear area is also an indication for dysfunctions in regular cell growth and division. Nuclear DNA was PI stained and analyzed by flow cytometry. Here, a marginal cell cycle delay at G_0_/G_1_ phase was observable. Since flow cytometric analysis of PI stained cells cannot distinguish between G_0_ (quiescence) and G_1_ phase [36], relating to the Ki67 staining results, it is likely that more knock out cells rest in G_0_ phase. One key regulator of G_0_/G_1_- S transition is Cyclin D1. Consistent to cell cycle analysis the expression of Cyclin D1 is decreased in eEF2K knock out cells. The peak shift to higher PI fluorescence intensities of around 2-fold reflects the double DNA content of knock out cells. It is reported, that hepatocytes are prone to increase their ploidy in terms of DNA damage [37] and cellular stress factors e.g. oxidative stress [38]. Also a hyperactivation of ERK1/2 is reported to promote aneuploidy by polyploidization [39]. However, the mechanisms how the absence of the eEF2 kinase impairs this pathway remain unclear and have to be subject of future studies.

Given that in embryonic fibroblasts of eEF2K^-/−^ mice this change in cellular phenotype is not reported [40, 41], this phenomenon could be cell type specific and likely would not occur within treatment of HCC. Yao et al. proposed C-terminal eEF2 fragments located in the nucleus promote abnormal nuclear morphology and polyploidy [42]. However, we immunohistochemically stained formalin-fixed control and eEF2K^-/−^ cells with an antibody against C-terminal eEF2 and no nuclear staining was observable (Supplementary data).

In summary we could show that eEF2 and peEF2(T56) are overexpressed in HCC-tissues and have a prognostic value for overall survival of HCC-patients. Additionally, we could demonstrate that eEF2 kinase exhibits a 4-5 fold increased activity in HCC tissue. Knocking out eEF2 kinase in tumor cells results in less proliferative cells and a decreased growth rate, though the increased translation leads to augmentation of cell size. Concluding these results, we propose eEF2 kinase being a possible and valuable drug target for HCC therapy.

## MATERIALS AND METHODS

### Patients

Informed consent was obtained from every patient and the study protocol conforms to the ethical guidelines of the 1975 Declaration of Helsinki. The local ethics committee approved the study (#15–6230-BO). Clinicopathological patient data is shown in Table 1. The tissue for TMAs and proteome-based studies was collected from 1999 until 2005 and in 2011/2012, respectively. Only patient without prior treatment were included.

### Phosphoprotein enrichment

Pierce Phosphoprotein Enrichment Kit (Pierce Biotechnology, Rockford, IL, USA) was used for enrichment of phosphoproteins. Samples were treated according to the manufacturer's protocol. Briefly, tissue samples were homogenized in lysis buffer containing 0.25% CHAPS (3-[(3-Cholamidopropyl)dimethylammonio]-1-propanesulfonate) and HALT protease- and phosphatase inhibitors (Pierce Biotechnology, Rockford, IL, USA). 4 mg of total protein were applied to phosphoprotein enrichment columns. Bound phosphoproteins were washed 3 times with lysis/binding/wash buffer (+0.25% CHAPS). Phosphoproteins were eluted using elution buffer (+0.25% CHAPS) and concentrated by vacuum concentration.

### 2D DIGE experiments

Protein labeling, 2D electrophoresis, scanning, image analysis, and statistics as well as digestion and peptide protein identification using mass spectrometry were performed as recently described [43].

### Immunohistochemical staining

Briefly, specimens were fixed overnight in 4% paraformaldehyde, dehydrated, and embedded in paraffin. Sections (1 μm) were pretreated with citrate buffer pH 6.0 for 30 min at 98°C. Primary antibodies used for immunohistochemistry are listed in Supplementary Table 2. Corresponding secondary enzyme-labeled antibodies were used in combination with the appropriate detection system. Sections were counterstained hematoxylin. Evaluation using a semi quantitative scoring system including number of positive cells (1: 1-5%; 2: 6-10%; 3: 11-50%; 4: >50%), staining intensity (1: weak, 2: medium, 3: intense) and the respective sum was performed by two independent experienced pathologists (HAB, HR). Statistical analyses were performed using Wilcoxon signed-rank. Survival analysis was calculated by Kaplan-Meier method and log-rank test. Immunohistochemical staining of cell culture was done on cells grown in chamber slides (Eppendorf, Hamburg, Germany) stained with an antibody against Ki67. Immunohistochemically stained cells were analyzed regarding cell and nucleus area by morphometric measurements with help of CellSens dimension 1.9 software (Olympus life science, Tokyo, Japan). Mann-Whitney *U* test was used for statistical evaluation.

### Western blotting

Lysates were prepared in lysis buffer (T-PER lysis buffer containing HALT protease- and phosphatase-inhibitors; Pierce Biotechnology, Rockford, IL, USA), separated by sodium dodecyl sulfate polyacrylamide gel electrophoresis and transferred to nitrocellulose membranes (Whatman, part of GE Healthcare, Munich, Germany). Membranes were blocked with 5% skimmed milk/TBS pH7.6/0.1% Tween20 for 1 h and incubated with primary antibodies (Supplementary Table 2) overnight. Corresponding horseradish-peroxidase-labeled anti-rabbit immunoglobulin G (1:15000; Cell Signaling Technology, Danvers, MA, USA) was then added for 60 min at room temperature. Finally, enhanced chemiluminescence (Pierce Biotechnology, Rockford, IL, USA) was used to visualize the results. Expression levels were analyzed by densitometry and normalized to total protein staining of the according lanes.

### eEF2 kinase assay

Lysates from liver tissue were prepared using lysis buffer (0.5% (v/v) NP-40, 150 mM NaCl, 1 mM CaCl_2_, 25 mM Na_4_P_2_O_7_, 50 mM β-glycerol phosphate disodium salt, 2 mM EDTA, 2 mM EGTA, 25 mM Tris, pH8.0, 10% (v/v) glycerol, 10μg/ml soybean trypsin inhibitor, 1 mM benzamidine, 1 mM PMSF, 50 mM NaF, 0.1 mM Na_3_VO_4_, 0.002% (w/v) NaN_3_). EEF2 kinase was immunoprecipitated using eEF2K antibodies (5 μl/mg lysate, Cell Signaling Technology, Danvers, MA, USA) bound to Protein A sepharose beads and with gentle rotation for 2h at 4°C. Beads were washed three to four times in phosphorylation buffer containing 50 mM Hepes (pH7.4), 10 mM MgCl_2_ and 1 mM CaCl_2_. For the kinase assay myelin basic protein (MBP; 1μg/sample; Sigma-Aldrich, Steinheim, Germany), Calmodulin (2.5μg/sample; Sigma-Aldrich), 10 μM ATP and [γ-^32^P]ATP (0.5-0.75μCi/sample; Hartmann-Analytic, Braunschweig, Germany) were added to immunoprecipitated eEF2 kinase. Unspecific kinase activity was determined by addition of the eEF2 kinase inhibitor NH125 (3 μM, Calbiochem (part of Merck KGaA, Darmstadt, Germany)) to indicated samples. After 20 min at 30 °C, the reaction was stopped by the addition of Laemmli buffer. Proteins were separated by SDS-PAGE and phosphorylation of MBP was quantified by PhosphoImager analysis. Protein amounts of immunoprecipitated eEF2K were controlled by Western blot analysis. Statistical evaluation was done using Wilcoxon signed-rank test.

### Next generation sequencing

Multiplex PCR and purification were performed with the GeneRead DNAseq Custom Panel, GeneRead DNAseq Panel PCR Kit V2 (Qiagen, USA) and Agencourt® AMPure® XP Beads (Beckmann, USA) on 24 randomly chosen NT and T tissue pairs. The library preparation was performed with NEBNext Ultra DNA Library Prep Set for Illumina (New England Biolabs, Ipswich, MA, USA), according to the manufacturer's recommendations by using 24 different indices per run. The pooled library was sequenced on MiSeq (Illumina; Reagent Kit V2; 2×150 bases paired-end run) and analyzed by Genomics Workbench (CLC Bio, Qiagen, USA). For read-mapping the *eEF2K* gene (NCBI-database) preserved as a reference.

For targeted sequencing a customized *eEF2K*-panel was designed covering 100% of the gene (including UTRs). The CDS of the gene *eEF2K* was covered by a total of 88 amplicons. In the two runs an average coverage of approximately 8000x and 4500x was obtained.

### Cell lines

Human hepatocellular carcinoma cell line JHH5 [44, 45] (JCRB1029, JCRB cell bank, Japan) were cultured in William's medium E with 10 % FCS and 1% penicillin/streptomycin in a 5 % CO_2_ atmosphere at 37°C.

### CRISPR/Cas9-mediated knock out

Cells were transfected via electroporation (Neon transfection system, Life technologies, CA, USA) with the following parameters: Pulse voltage: 1200 V, pulse width: 50 ms, pulse number: 1, 500000 cells/ml. For Knock-out the GeneArt® CRISPR nuclease Vector with OFP reporter (Life technologies; CA, USA) containing a 20bp guideRNA (top strand: 5’CTACATCGAGCCCGTAGACC 3’; bottom strand: 5’GGTCTACGGGCTCGATGTAG 3’) was used. For control cells no guiding RNA was inserted. The guideRNA was selected with the assistance of the CRISPR design tool from the Zhang laboratory at the Massachusetts Institute of Technology (http://crispr.mit.edu/) and targets exon 6 of the human *eEF2K* gene. Five different gRNAs were tested for Cas9 cleavage efficiency with GeneArt genomic cleavage detection kit (Life technologies, Carlsbad, CA, USA) as stated in the manufacturer's manual. Only the gRNA yielding the highest cleavage rate was chosen for further transfection. Transfected cells were single cell cloned into 96-well plates at 0,3 cells/well, checked for the expression of OFP, expanded to populations and analyzed by PCR amplifying this region of genomic DNA using primers flanking the target region in *eEF2K* [forward: GCTCAGGAAGAACAGGGAACACC, reverse: GGATTCACCATGGCCCTCTTTC] followed by TOPO-cloning and sequencing of multiple inserts.

To exclude Cas9 off-target activities, cleavage detection was performed for seven predicted off-target sites. No off-target effects were detectable (data not shown).

### Cell proliferation

To determine growth curves in three independent experiments cells were seeded out in 6-well plates in triplicates at 30 % subconfluence. After 24, 72, 120, 144 and 168 hours cells were removed from wells by trypsinization using 0.25 % Trypsin-EDTA and counted in a haemocytometer. Data was expressed as mean ± SD and student's t-test was performed for statistical analysis. Doubling times were calculated as following in log phase (24-168 h):

### Cell cycle analysis

Flow cytometric analysis of the cell cycle phase distribution of JHH5 cell lines was performed 24 hours after seeding. Cells were stained with propidium iodide (PI) as previously described [46].

Cell cycle phase distribution was analyzed using a Cytoflex 2.51 (Beckmann Coulter, Pasadena, CA, USA) flow cytometer and the CytExpert 1.2 software. Excitation and emission wavelength were set to 488 nm and 585/42 nm, respectively.

### Statistical analyses

SPSS version 19 (IBM) was used for analyses. A *P*-value less than .05 was considered as statistically significant.

## SUPPLEMENTARY MATERIALS FIGURES AND TABLES





## Abbreviations

CHAPS: 3-[(3-Cholamidopropyl)-dimethylammo nio]-1-propanesulfonate CRISPR= Clustered Regulatory Interspaced Palindromic Sequence
eEF2: eukaryotic elongation factor 2
ERK: Extracellular-signal Regulated Kinase
HBV: Hepatitis B-Virus
HCV: Hepatitis C-Virus
HSP90: Heat Shock Protein 90
MALDI-TOF-MS: Matrix-Assisted Laser Desorption/Ionization Time Of Flight Mass Spectrometry
MAP-kinase: Mitogen Activated Protein-kinase
mTOR: mammalian Target Of Rapamycin
NH125: 1-Benzyl-3-cetyl-2-methylimidazolium iodide
peEF2(T56): eEF2 phosphorylated at threonine 56
pERK1/2: phospho-p44/42 MAP-kinase
eEF2 kinase: Eukaryotic elongation factor-2 kinase
SDS-PAGE: Sodium Dodecyl Sulfate Poly Acrylamide Gel Electrophoresis
TMA: Tissue Micro Array
