# Supplementary material for: Eukaryotic elongation factor 2 is a prognostic marker and its kinase a potential therapeutic target in HCC
Source: Oncotarget. 2017 Jan 2;8(7):11950–62. doi: 10.18632/oncotarget.14447 (PMC5355317; doi:10.18632/oncotarget.14447)
Supplement: Supplementary file 1 [file oncotarget-08-11950-s001.pdf]

Eukaryotic elongation factor 2 is a prognostic marker and its kinase a potential therapeutic target in HCC

SUPPLEMENTARY FIGURES AND TABLES

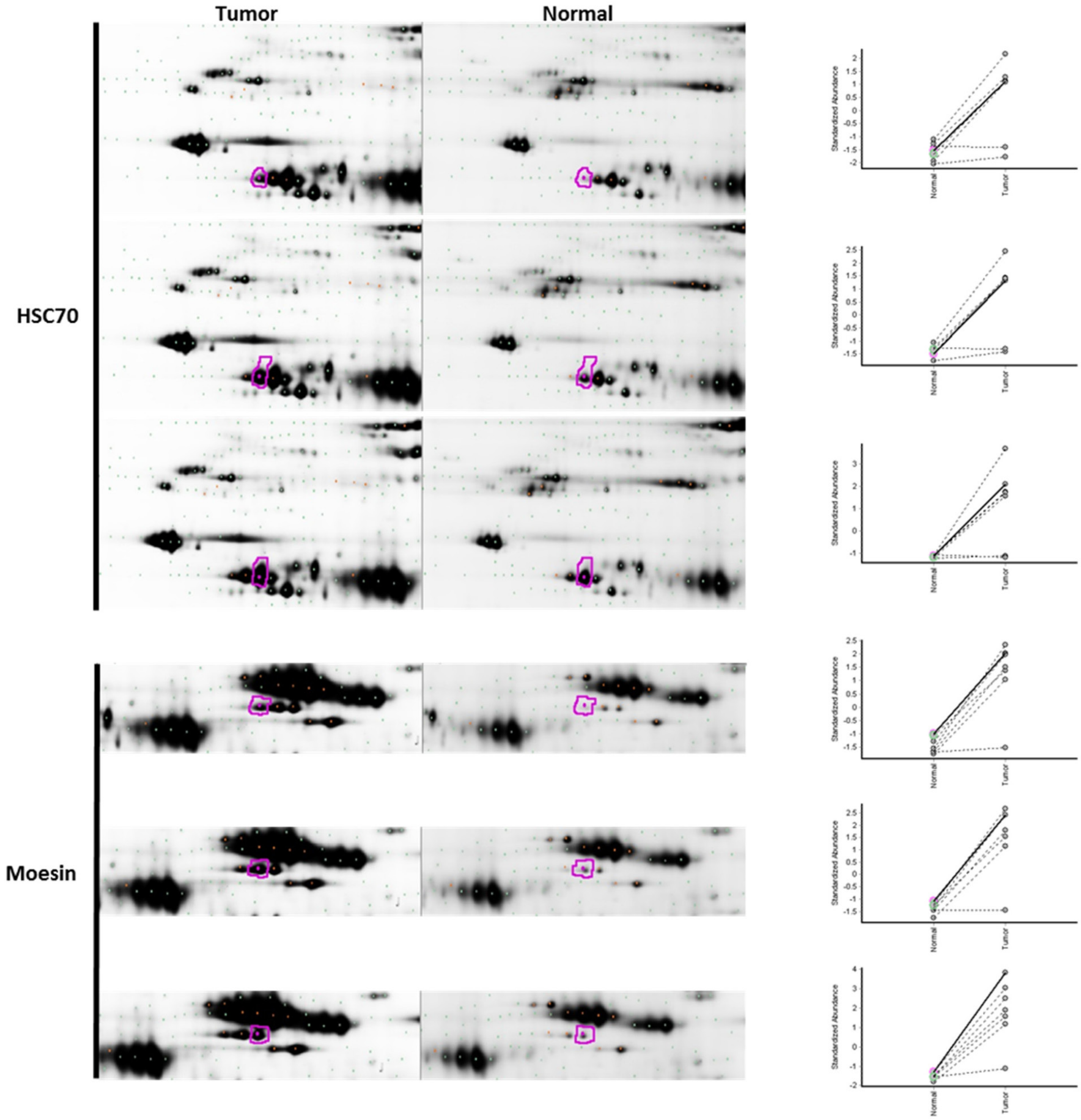

Supplementary Figure 1: The spots of the two candidate proteins HSC70 and Moesin resulting from 2D DIGE analysis of corresponding phosphoprotein-enriched non-tumorous and HCC liver tissue of 7 HCC patients. Representative spots (marked in pink) of HSC70 (spots 522, 524 and 531) and Moesin (spots 466, 467 and 468) in non-tumorous (normal, right part) and corresponding HCC (tumor, left part) liver tissue lysate, enriched for phosphoproteins, of one HCC-patient. The graph on the right shows the normalized expression levels of the corresponding phosphoprotein-enriched non-tumorous and tumorous liver lysates of all 7 HCC-patients, which were analyzed in this study.

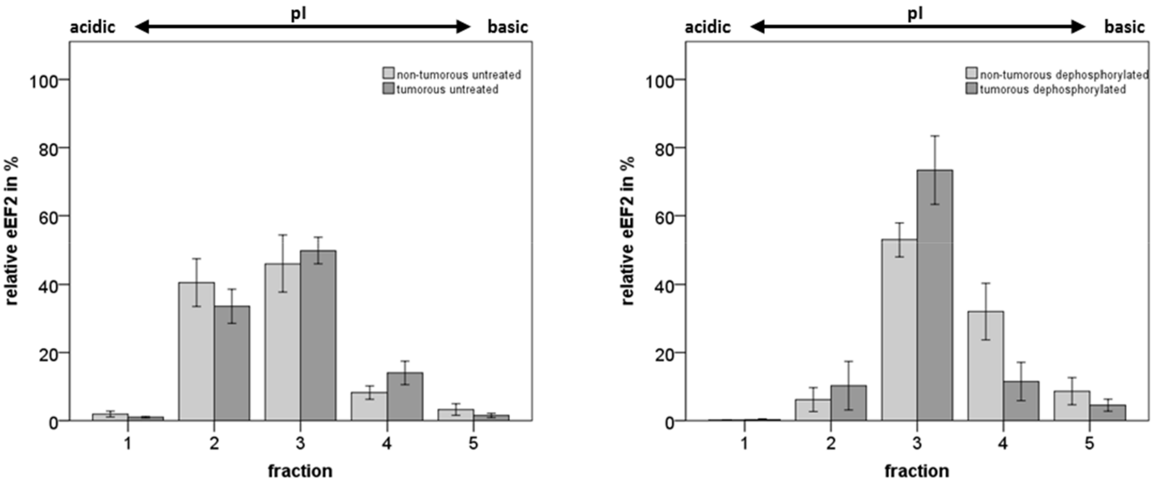

Supplementary Figure 2: Analysis of the general phosphorylation state of eEF2 in total protein lysates of non-tumorous and HCC liver tissues. Off-gel fractionation and subsequent immunoblotting of the obtained fractions with densitometric determination of the relative protein distribution in the fractions before and after treatment with lambda protein-phosphatase were performed. In general, after dephosphorylation a shift of the relative protein distribution in the fractions towards a more basic pI due to the loss of negatively charged groups could be observed for eEF2. The effect was detectable between untreated and dephosphorylated as well as between non-tumorous and tumorous lysates. Data shown are mean ± SE.

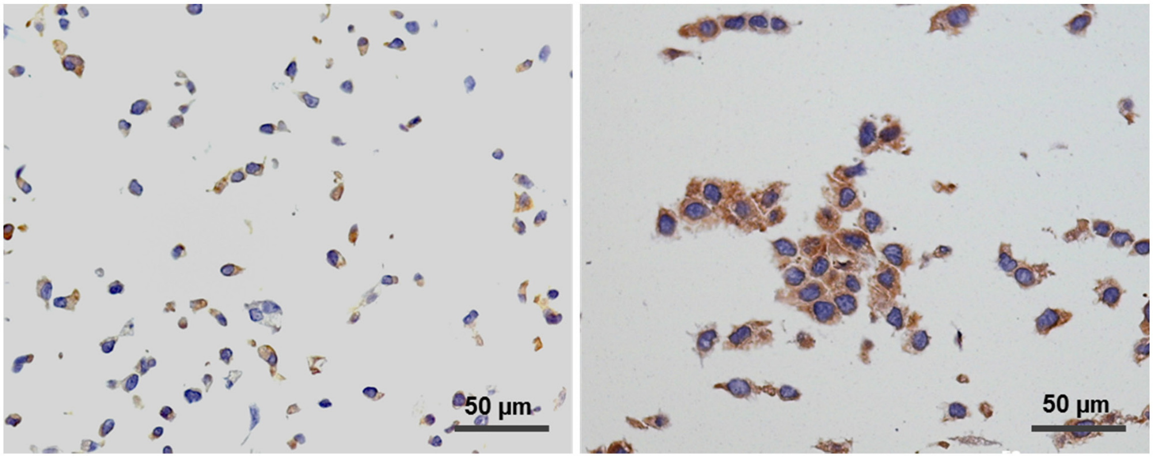

Supplementary Figure 3: Control cells (left panel) and eEF2K-/- cells (right panel) were harvested, formalin-fixed and pelleted in agarose. 1 µm specimens were immunohistochemically stained against C-terminal eEF2. The cytosolic staining is clearly visible in both cell types. No nuclear staining could be observable.

Supplementary Table 1: List of the quantified spots revealed by 2D DIGE and their MALDI TOF protein identification

See Supplementary File 1

Supplementary Table 2: Primary antibodies used in this study (* Immunohistochemistry, # Immunoblot)

Antigen	Delivery	Dilution	Application
eEF2	Abcam	1:400	IHC*
peEF2(T56)	Cell Signaling Techn.	1:200	IHC
HSC70	Abcam	1:75	IHC
Moesin	Abcam	1:500	IHC
pEzrin-Radixin-Moesin	Cell Signaling Techn.	1:25	IHC
Ki67	Acris	1:200	IHC
eEF2	Abcam	1:10000	IB#
peEF2(T56)	Cell Signaling Techn.	1:8000	IB
eEF2 kinase	Abcam	1:2000	IB
Cyclin D1	Cell Signaling Techn.	1:4000	IB
ERK1/2	Cell Signaling Techn.	1:5000	IB
pERK1/2 (T202,Y204)	Cell Signaling Techn.	1:4000	IB
pERK(T188)	Provided by K. Lorenz [36]	1:1500	IB
