# Supplementary material for: Eukaryotic elongation factor 2 is a prognostic marker and its kinase a potential therapeutic target in HCC
Source: Oncotarget. 2017 Jan 2;8(7):11950–62. doi: 10.18632/oncotarget.14447 (PMC5355317; doi:10.18632/oncotarget.14447)
Supplement: Supplementary file 2 [file oncotarget-08-11950-s002.docx]

Supplemental table 1: List of the quantified spots revealed by 2D DIGE and their MALDI TOF protein identification.

| **Spot Number** | **Protein Name** | **Fold Change** | **ANOVA** | **Known phospho-protein** |
| --- | --- | --- | --- | --- |
| 74 | CPS1 Isoform 2 of Carbamyol-phosphate sythate, mitochondrial | -2,14 | 0,011 |  |
| 133 | CPS1 Isoform 2 of Carbamyol-phosphate sythate, mitochondrial | 1,93 | 0,022 |  |
| 217 | EEF2 Elongation factor 2 | 1,89 | 0,000077 | x |
| 219 | EEF2 Elongation factor 2 | 2,1 | 0,014 | x |
| 220 | EEF2 Elongation factor 2 | 1,61 | 0,04 | x |
| 236 | ACO1 Cytoplasmatic sconitate hydratase | -2,4 | 0,000032 |  |
| 241 | ACO1 Cytoplasmatic sconitate hydratase | -1,76 | 0,0025 |  |
| 242 | ACO1 Cytoplasmatic sconitate hydratase | -2,61 | 0,00004 |  |
| 271 | ALDH1L1 Aldehyde dehydrogenase family 1 member L1 | -2,64 | 0,008 | x |
| 272 | ALDH1L1 Aldehyde dehydrogenase family 1 member L1 | -2,33 | 0,015 | x |
| 299 | HSP90B1 Endoplasmin | -1,6 | 0,02 | x |
| 389 | EZR Ezrin | 1,73 | 0,000023 | x |
| 394 | TF Serotransferrin | 2,37 | 0,00088 | x |
| 396 | TF Serotransferrin | 2,52 | 0,00043 | x |
| 397 | TF Serotransferrin | 2,52 | 0,00043 | x |
| 398 | TF cDNA FLJ54029, highly similar to Serotrasferrin | 2,11 | 0,0032 | x |
| 470 | MSN Moesin | 2,12 | 0,0029 | x |
| 471 | MSN Moesin | 2,43 | 0,0015 | x |
| 513 | MSN Moesin | 3,15 | 0,00024 | x |
| 516 | C4A Uncharaterized Protein | 2,28 | 0,00055 |  |
| 522 | C4A Uncharaterized Protein | 2,61 | 0,000056 |  |
| 524 | C3 Complement C3 | 3,1 | 0,00015 | x |
| 531 | C3 Complement C3 | 3,14 | 0,00015 | x |
| 652 | HSPA8 Uncharacterized Protein | 1,73 | 0,02 | x |
| 693 | HSPA8 Isoform 1 of Heatshock cognate 71 kDa protein | 1,81 | 0,0087 | x |
| 701 | HSPA8 Isoform 1 of Heatshock cognate 71 kDa protein | 2,01 | 0,0095 | x |
| 702 | UDGH UDP-glycose 6-dehydrogenase | -1,76 | 0,0069 | x |
| 705 | CES1 Isoform 1 of Liver corboxylesterase 1 | -1,89 | 0,018 |  |
| 706 | CES1 Isoform 1 of Liver corboxylesterase 1 | -1,82 | 0,03 |  |
| 714 | CES1 Isoform 1 of Liver corboxylesterase 1 | -1,68 | 0,045 |  |
| 721 | CES1 Isoform 1 of Liver corboxylesterase 1 | -1,83 | 0,024 |  |
| 730 | CES1 Isoform 1 of Liver corboxylesterase 1 | -1,84 | 0,029 |  |
| 754 | FTCD Isoform A of Formimidoyltransferase-cyclodeaminase | -2,53 | 0,0097 | x |
| 757 | GLUD1 Uncharaterized Protein | -1,57 | 0,03 | x |
| 761 | FGG Isoform Gamma-A of Fibrinogen gamma chain | -1,89 | 0,016 | x |
| 763 | FGF12 16 kDa Protein | -2,04 | 0,00034 | x |
| 848 | GATM Isoform 1 of Glycine amidinotransferase, mitochondrial | -1,95 | 0,00097 | x |
| 886 | GATM Isoform 1 of Glycine amidinotransferase, mitochondrial | -1,94 | 0,03 | x |
| 932 | ERP44 Endoplasmatic reticulum resident protein 44 | 1,73 | 0,0026 |  |
| 974 | UPB1 UPB1 Protein | -1,8 | 0,046 |  |
| 1046 | ADH1C Alcohol dehydrogenase 1c | 2,55 | 0,0054 |  |
| 1087 | RAB30 Uncharacterized Protein | -2,08 | 0,0073 | x |
| 1092 | ACADSB Short/branched chain specific acyl-CoA dehydrogenase, mitochondrial | -2,58 | 0,00024 |  |
| 1100 | FBP1 Fructose-1,6-bisphosphatase 1 | -3,37 | 0,0036 |  |
| 1109 | FBP1 Fructose-1,6-bisphosphatase 1 | -3,11 | 0,00047 |  |
| 1110 | GAPDH Glyceraldehyd-3-phosphate dehydrogenase | 3,09 | 0,035 | x |
| 1207 | AKR7A3 Aflatoxin B1 aldehyde reductase member 3 | -1,89 | 0,012 |  |
| 1290 | PBLD Phenazine biosynthesis-like domain-containing protein | -4,57 | 0,000001 |  |
| 1291 | PNP cdNA, highly similar to Purine Nucleoside Phosphorylase | -2,09 | 0,00012 |  |
| 1357 | MKI67 Isoform short of Antigen Ki-67 | -1,84 | 0,023 | x |
| 1371 | CA2 Carbonic anhydrase | -1,93 | 0,0031 |  |
| 1384 | ERP29 Endoplasmatic reticulum resident protein 29 | -1,75 | 0,00097 | x |
| 1493 | GSTK1 Isoform 1 of Glutathione S-Transferase kappa 1 | 1,83 | 0,0074 |  |
| 1556 | NME1;NME2 Uncharaterized Protein | 2,23 | 0,00099 |  |
